# Exploratory Report of Wild Boar Surveillance and Epidemiological Course of African Swine Fever Outbreak; Case in the Republic of Korea From 2019 to 2023

**DOI:** 10.1155/tbed/3538366

**Published:** 2025-12-04

**Authors:** Jusun Hwang, Eunsol Kim, Jeonghyuk Kim, Jicheol Kim, Yeonji Kim, Sanggeun Lee, Yongkwan Kim, Hyunjun Cho, Sungin Ji, Jisoo Kim, Sanghyun Lee, Kidong Son, Weon-Hwa Jheong

**Affiliations:** ^1^ Wildlife Disease Response Team, National Institute of Wildlife Disease Control and Prevention (NIWDC), 1 Songam-gil, Gwangsan-gu, Gwangju, 62407, Republic of Korea

## Abstract

In October 2019, the first cases of African swine fever (ASF) were confirmed in wild boar in the Republic of Korea (ROK). Since then, ASF has continued to spread throughout the country, particularly among wild boar populations, despite intensive efforts to contain the outbreak. Our objective was to assess the results of current ASF surveillance in wild boars and identify various risk factors related to ASF outbreak in ROK. Between September 2019 and June 2023, a total of 122,078 wild boar samples were submitted, with 3135 tested as positive to ASF infection. Among them, 90.6% (2839/3135) of ASF‐positive cases were detected from carcasses. Within the carcass samples, ASF prevalence showed an increase from 5.98% (95% confidence interval [CI] 4.47–7.81) to 37.15% (95% CI 34.12–40.27) through years 2019 and 2023, respectively. Geographically, from the demilitarized zone (DMZ), the region where ASF was first detected in wild boars, the disease has spread toward the east and south direction across the Baekdu mountain range, which is a continuous forest habitat. The movement of the ASF outbreak region between years has been relatively constant for the last 45 months, except for between 2020 and 2021 due to rapid spread during the year 2021. From the ASF‐specific antibody assay, only two wild boars were seropositive out of 17,275 serum samples. This is the first study presenting results and patterns of wild boar surveillance and ASF virus prevalence changes in the ROK during 2019–2023. The study showed that output of surveillance and control effort, as well as the epidemiologic course of ASF in ROK is potentially associated with various spatial and anthropogenic factors, with subsets of factors more linked with specific circumstances of ROK. A better understanding regarding the past and current status of the disease will contribute to improving control measures required for preventing further spread of the ASF virus in the Korean peninsula.

## 1. Introduction

African swine fever (ASF) is a highly transmissible hemorrhagic disease caused by a dsDNA virus, which is a member of the *Asfivirus* genus. The virus was first identified in 1921 in Kenya, and has been mainly found in the African continent, in natural hosts, including *Ornithodoros* soft ticks and African *suidae* species, such as bush pig and warthogs [1]. Nevertheless, the ASF virus of genotype I was first introduced to European countries through Portugal in 1957. Unlike African *suidae* species, which experienced long‐term coevolution with the virus, acquiring innate immunity and remaining asymptomatic after ASF infection, almost 100% mortality was observed in domestic pigs and Eurasian wild boars [2, 3]. Although the first introduction to European continent was almost eliminated in 1995, the second introduction of the ASF virus genotype II was reported in 2007, in Georgia, which subsequently spread in various directions, reaching western European countries to the west, while crossing throughout Russia to China, east and southeastern Asian countries [4].

In wild animals, the epidemiology of disease tends to be tightly linked with the ecology of its main host species, which in the case of ASF is the wild boar in many affected Eurasian countries. The major transmission mode of the ASF virus in wild boars is through direct contact with infected carcass/environmental virus contaminants, or contact with live infected animals, particularly within groups, given their group‐living social structure [5]. The contact heterogeneity among wild boar individuals together with the territoriality and high fatality of ASF infection, which hampers mobility of wild boars, are potential reasons for slow spread (1–2 km/month) of the disease in undisturbed wild boar populations [6, 7]. As in other wildlife‐associated disease management, surveillance and intervention of ASF in wild boar is extremely difficult and those that are experiencing long‐term outbreak are sacrificing massive numbers of both domestic pigs and wild boar, potentially causing detrimental effect on the endemic ecosystem in addition to economic losses.

In the case of the Republic of Korea (ROK), ASF virus is suspected to have spread through Russia and China, while the involvement of North Korea is undetermined [8]. The first outbreak in China was reported in 2018, and the North Korea in May 2019, followed by first detection of ASF in pig farms and wild boar in the fall of 2019 in the ROK [9]. In the ROK, the majority of ASF virus has been detected in wild boar, which is similar to many European countries, where the disease is mainly carried and considered self‐sustained by the wild boar population, with farm outbreaks concentrated in small holdings with low biosecurity [10]. Therefore, the majority of the ASF control measures applied in EU countries were adopted in the ROK, with priority in carcass search, population control, and construction of fences. An incentive for hunting wild boar was applied nationwide immediately after the first outbreak, with the allowance of hunting activity opened year‐round. Fences of various lengths and structures were constructed for the purpose of either containing the outbreak or decelerating the spread. The carcass search team was mainly composed of local residents and hunters. Military personnel were also deployed for carcass searches (1) near the Korean demilitarized zone (DMZ) region, where there are areas that nonmilitary personnel have restricted access, and (2) on occasions when a long‐distance “jump” of ASF outbreak occurred, and a systematic carcass search was critically urgent in the limited area. Lastly, trained search dogs were mobilized for carcass search as a pilot project from July 2022. Dogs were strategically used at locations where immediate elimination of fresh positive carcasses was required, but were limited due to rough terrain, which minimizes the efficiency of search effort.

Despite substantial efforts invested in the eradication of the disease since its first detection, ASF is still present in the wild boar population of the ROK, propagating to southern regions. Initial control of the ASF outbreak in Korean wild boars has been exceptionally difficult due to various reasons. For example, the ROK had limited access to the information regarding ASF outbreak and wild boar population in the northern region of the DMZ, which is the area where the first wild boar ASF case was found in the ROK [11]. Without necessary data, it has been difficult to proactively predict and invest in control measures in higher‐risk regions near the border. In addition, sporadic minefields distributed throughout the vicinity of the DMZ imposed major restrictions on performing thorough carcass search activity and elimination of wild boars within the outbreak region. As the ASF front wave moved further south, two new risk factors are suspected to have emerged. These include anthropogenic “jump” events causing long‐distance transmission, and difficulties in motivating hunters to comply with recommended field practice‐factors reported to limit the effectiveness of ASF control measures in other countries [8, 12, 13]. To fill the knowledge gaps regarding the epidemiology of ASF in wild boar population of the ROK and update current interventions accordingly, we analyzed comprehensive ASF surveillance data from the ROK and explained the course of disease over time.

## 2. Methods and Materials

### 2.1. Surveillance and Sample Collection

A surveillance program targeting ASF in wild boar was initiated after the first detection of ASF from a wild boar carcass in October 2019. From then till the summer of 2022, wild boar samples of (1) all found carcasses, (2) animals all hunted/trapped within cities/counties that experienced an ASF outbreak,and (3) 10% of wild boars hunted/trapped in cities/counties free of ASF were tested for the ASF virus. The extent of “outbreak region” gradually expanded with the evolution of epidemiological situation. Since July 2022, a new surveillance strategy, which mandates testing all wild boar samples, regardless of region or collection source (hereafter “nationwide surveillance strategy”), was implemented to detect unforeseen “jump” cases that may be occurring in regions where ASF has not been detected before.

Wild boar surveillance focused primarily on the molecular detection of viruses, with antibody testing performed when serum samples were available. Samples were obtained from three main sources: carcasses reported by the public or detected by search teams, hunted animals, and trapped individuals. Trapping was conducted using box traps and drop‐net systems, mostly in areas near outbreak regions where hunting with dogs was restricted. The most common sample types were blood and muscle, although skin and bone marrow were also collected from carcasses. Fresh blood samples, when available, were centrifuged to separate serum for further testing. For all wild boars, the following information was collected from the field: sex and estimate of body mass (when possible), date, coordinate of the location, address, and photo. All collected samples and information were sent to the National Institute of Wildlife Disease Control and Prevention (NIWDC) and tested for the presence of ASF virus DNA and antibodies against ASF virus using molecular and serological methods. Data used in this study includes the analysis results of samples collected from September 1, 2019 to June 3, 2023.

### 2.2. Laboratory Analysis

Molecular detection of ASF virus was conducted through DNA extraction, real‐time polymerase chain reaction (PCR) for screening, and conventional PCR for confirmation analysis. DNA was extracted from the blood or supernatant of homogenized tissues using a Maxwell viral total nucleic purification kit (Promega, Madison, WI, USA), and qRT‐PCR (Applied Biosystems; Waltham, MA, USA) was performed using the VetMAX ASFV Detection Kit (Thermo Fisher Scientific; Waltham, MA, USA) according to the manufacturer’s instructions. Conventional PCR targeting for the B646L gene was subsequently performed for samples with threshold cycle (Ct) value below 35 in RT‐PCR. Primer sets used for conventional PCR were PPA1(5′‐AGTTATGGGAAACCCGACC C‐3′), PPA2 (5′‐CCCTGAATCGGAGCATCCT‐3′), P72D (5′‐GTACTGTAACGCAGCA CAG‐3′), and P72U (5′‐GGCACAAGTTCGGACATGT‐3′) [14].

Antibody testing was performed using a commercial ID Screen ASF Indirect (IDvet, Grabel, France) ELISA kit, which detects antibodies against p32, p62, and p72. All tests were conducted adhering to the manufacturer’s instructions. Test results were read and interpreted using spectrophotometry at 450 nm wavelength.

### 2.3. Data Analysis

For calculating wild boar hunting bag data, the total number of hunted/trapped wild boar data was available at the province level, and hence were divided by the area of each province (in km^2^). Prevalence estimations and confidence intervals (CIs) were calculated by year and sample source using Clopper and Pearson [15]. Multiple logistic regression analyses were performed to evaluate factors associated with ASF virus detection status based on PCR assay (positive vs. negative). The first model tested the effect of sample source (carcass, hunted, trapped) using all the tested samples. The second model tested biological factors (sex and age) using only samples with complete metadata, as many data lacked information on the sex and/or age of the animal tested. Age was conservatively classified into two groups based on their estimated body weight, with individuals body weight below 30 kg as piglets and over 30 kg as adults. Model selection for logistic regression was conducted using a stepwise procedure based on Akaike’s information criterion (AIC), and the final model was chosen as the most parsimonious fit (AIC = 20,889). Model fit was further evaluated using McFadden’s pseudo‐*R* ^2^ (0.006). The interaction term (sex × age) was retained in the final model, as it significantly improved model fit (*p* = 0.009).

The third logistic regression analysis was conducted using only carcass samples to test for the association between ASF results with temporal variables, year, and month. To capture the cyclic nature of seasonal variation, month was transformed using sine and cosine terms (sin[2*π*·month/12] and cos[2*π*·month/12]), a common approach for modeling periodic effects in epidemiological time series [16, 17]. Age and sex were not tested as many of the carcass samples were in process of decomposition and did not provide confident information regarding either factor. The initial full model included interactions between sine(month), cosine(month), and year, and model selection was performed using AIC. However, the interaction terms were not supported by model selection (ΔAIC > 2) and none were statistically significant (all *p* > 0.2), indicating that the overall seasonal pattern was consistent across years. Therefore, we retained the simpler model including only the main effects of sine(month), cosine(month), and year as a fixed effect. After fitting the models, Pearson residuals were extracted and examined using the autocorrelation function (ACF) and partial ACF (PACF) plots to assess any remaining temporal autocorrelation. Statistical significance was defined as *p* < 0.05. Model estimates are reported as odds ratios (ORs) with corresponding 95% CIs.

Spread of disease was analyzed and visualized in two complementary ways: (i) expansion of the affected area, and (ii) interannual distance between ASF‐positive cases. In the first method, the spatial expansion of ASF‐positive areas was defined at the administrative township level (“eup” or “myeon”). When a new ASF‐positive case was confirmed in a previously unaffected township, that unit was considered newly affected, and its area was added to the cumulative ASF‐affected area. Although township sizes vary, most are relatively small and comparable nationwide, with more than half (≈52%) between 1–30 km^2^ and about two‐thirds (≈67%) within 0.5–50 km^2^, while only a few mountainous units exceeded 70 km^2^ (Figure S1). For the second approach, we estimated yearly front displacement by calculating all pairwise distances between ASF‐positive locations detected in the last month of each year (December for 2019–2022; April–May for 2023 to balance sample size) and those of the following year. The median and maximum of these distances were divided by 12 to yield an estimated monthly movement rate. This direct point‐to‐point method provides a simple and reproducible way to quantify front displacement from observed case coordinates.

To characterize the altitudinal shift of ASF detections, we calculated the mean elevation within a 1 km buffer around each ASF‐positive location. Elevation values were derived from a 30‐m resolution DEM using zonal statistics in QGIS. The 1 km buffer was chosen as a biologically reasonable scale.

All analyses of results were performed using R (https://r-project.org/, accessed on June 25, 2023). Creating plots and figures illustrating the yearly distribution of ASF cases on maps and calculating the size of ASF‐affected townships were performed using QGIS 3.22.1(QGIS Geographic Information System. QGIS Association. http://www.qgis.org, accessed on January 20, 2022).

## 3. Results

### 3.1. Descriptive Analysis of ASF Surveillance Data

**Figure 1:**
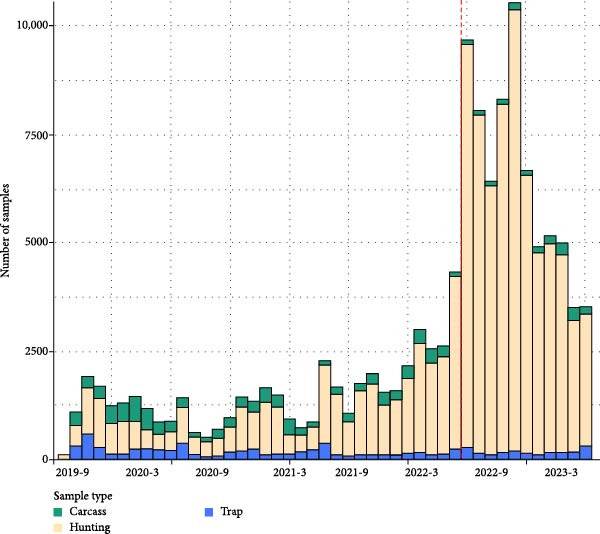
Temporal pattern of surveillance sample collection.  ^∗^The red dotted line shows the time point when “nationwide surveillance strategy” was initiated.

In the period of this study, a total of 122,078 wild boar samples were collected and analyzed for wild boar surveillance of ASF in the ROK. Among them, 9685 (7.9%) were obtained from wild boar carcasses, while 104,619 (85.7%) and 7,774 (6.4%) samples were collected from hunted and trapped wild boars, respectively. The number of samples gradually increased from 2019 to 2021 and surged in 2022 (from 17,136 samples in 2021 to 65,785 in 2022), with the implementation of “nationwide surveillance strategy” as described in the method section (Figure 1, Table 1). Nevertheless, the sample size of carcasses did not show a dramatic change in yearly pattern, as all carcass samples were submitted for testing irrespective of adopting the “nationwide surveillance strategy” (Table 2). The number of carcasses or trapped wild boar samples was highest in 2020. Regarding seasonal patterns, in all years, the number of total samples tested was largest in the fall (September–November) months (Table 3). When separated by its source, the number of samples from carcasses and trappings was highest in spring, whereas the number of hunted wild boars was highest in fall (Table 3).

**Table 1:** Surveillance samples collected from 2019–2023.

Category	Classification	Number of samples (%)
Year	2019	4760 (3.9)
	2020	12,361 (10.1)
	2021	17,136 (14.0)
	2022	65,785 (53.9)
	2023	22,011 (18.0)

Season	Spring (March–May)	26,344 (21.6)
	Summer (June–August)	29,981 (24.6)
	Fall (September–November)	35,204 (28.8)
	Winter (previous year December–February)	30,549 (25.0)

Type	Carcass	9685 (7.9)
	Hunt	104,619 (85.7)
	Trap	7774 (6.4)

Sex	Female	56,291 (46.1)
	Male	55,751 (45.7)
	Unknown	10,036 (8.2)

Age	Piglet (~30 kg)	40,976 (33.6)
	Adults (over 30 kg)	67,786 (55.5)
	Unknown	13,316 (10.9)

**Table 2:** Number of ASF‐positive and tested wild boar samples by year and sample source (all values are based on tested samples).

Year	Number of positives/number of total samples				Percentage of positives (CI 95%) from total sample size (%)			
	Total	Carcass	Capture		Total	Carcass	Capture	
			Hunting	Trap			Hunting	Trap
2019	55/4760	50/836	4/2773	1/1151	1.16 [0.87–1.50]	5.98 [4.47–7.81]	0.14 [0.04–0.37]	0.09 [0.00–0.48]
2020	856/12,361	814/3287	23/6953	19/2121	6.93 [6.48–7.39]	24.76 [23.30–26.28]	0.33 [0.21–0.50]	0.90 [0.54–1.40]
2021	965/17,136	849/2454	91/12,882	25/1800	5.63 [5.29–5.99]	34.60 [32.71–36.52]	0.71 [0.57–0.87]	1.39 [0.90–2.04]
2022	880/65,785	761/2106^a^	114/61,848	5/1831	1.34 [1.25–1.43]	36.13 [34.08–38.23]	0.18 [0.15–0.22]	0.27 [0.09–0.64]
2023	379/22,036	365/1002^a^	10/20,163	4/871	1.71 [1.55–1.90]	37.15 [34.12–40.27]	0.05 [0.02–0.09]	0.46 [0.13–1.17]
Total	3135/122,078	2839/9685	242/104,619	54/7774	2.57 [2.48–2.66]	29.29 [28.39–30.21]	0.23 [0.20–0.26]	0.69 [0.52–0.91]

^a^Among carcass samples, 48 samples were found by search dogs (24 samples each in 2022 and 2023). Among them, 12 samples in 2022 (50%) and 17 samples in 2023 (70.8%) were positive for ASF.

**Table 3:** Seasonal virus prevalence of ASF stratified by sample source and age.

Season	Sample source			Age (piglet: ~30 kg, adult: over 30 kg)			Total
	Carcass	Hunting	Trap	Piglet	Adult	Unknown	
Winter	38.93 (1161/2982)	0.32 (82/25,822)	1.09 (19/1745)	3.77 (342/9064)	3.92 (694/17,699)	5.97 (226/3786)	4.13 (1262/30,549)
Spring	31.91 (1152/3610)	0.28 (57/20,644)	0.33 (7/2090)	3.90 (251/6433)	4.45 (745/16,747)	6.95 (220/3164)	4.62 (1216/26,344)
Summer	19.02 (218/1146)	0.23 (62/26,820)	1.09 (22/2015)	1.09 (149/13,711)	0.71 (101/14,323)	2.67 (52/1947)	1.01 (302/29,981)
Fall	15.82 (308/1947)	0.13 (41/31,333)	0.31 (6/1924)	0.91 (107/11,768)	0.82 (156/19,017)	2.08 (92/4419)	1.01 (355/35,204)
Total	29.31 (2839/9685)	0.23 (242/104,619)	0.69 (54/7774)	2.07 (849/40,976)	2.50 (1696/67,786)	4.43 (590/13,316)	2.57

*Note:* Values in parentheses indicate the number of positive cases and the total number of samples within each corresponding subgroup.

The number of samples between the two sexes was similar (female *N* = 56,291, male *N* = 55,751, unknown *N* = 10,036), regardless of sample type (Table S2). Regarding age, the largest proportion of samples were adults (55.5%), followed by piglets (33.6%) and age‐unidentified samples (10.9%). When analyzed by sample source, 54.0% (5228 out of 9685) and 43.7% (4230 out of 9685) of carcass samples lacked information on sex or age, respectively (Table S2).

**Figure 2:**
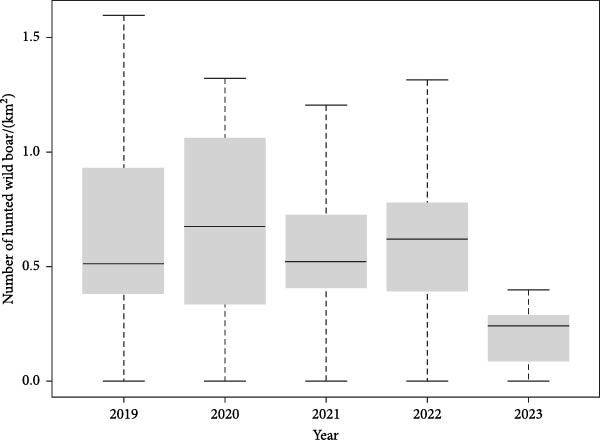
Numbers of hunted wild boar/km^2^ during 2019–2023.

Since not all hunted animals were tested for ASF before the implementation of “nationwide surveillance strategy”, the temporal pattern of wild boar hunting bag size (including both hunted and trapped boars) differs from the size of surveillance samples. Overall, the number of nationwide hunting bags was the largest in 2019, reaching 99,920 animals (0.99 km^2^), with more than 49,000 boars hunted between October and December, immediately after the outbreak of ASF. Since then, hunting bag size decreased constantly, with 21,034 animals hunted/trapped in the year 2023 (until 2^nd^ of June) within the study period (Figure 2).

### 3.2. ASF Prevalence in Wild Boar Populations

The overall prevalence of ASF virus in wild boars during the study period was 2.57% (carcasses, hunted and trapped), being highest in 2020 (6.93%) and lowest in 2019 (1.16%). Nevertheless, comparisons between earlier years (2019–2021) and later years (2022–2023) should be made with caution, because the sample size rose sharply during 2022 due to the implementation of the “nationwide surveillance strategy”. In relation to sample types, 90.6% (2839/3135) of positive samples were from carcasses, while 7.7% (242/3135) and 1.7% (54/3135) of positives originated from hunted and trapped wild boars, respectively. It should be noted that these proportions represent the relative contribution of each sample source among all ASF‐positive cases. Meanwhile, within each sample type, the prevalence was 29.31% (2839/9685) for carcasses, 0.23% (242/104,619) for hunted animals, and 0.69% (54/7774) for trapped animals (Table 2). Accordingly, the odds of ASF detection in trapped samples were approximately three times higher than in hunted samples (OR ~3.0; 95% CI: 2.0–4.6). All prevalence values were calculated based on the tested samples.

Seasonally, the ASF virus prevalence was 4.13% in winter and 4.62% in spring, while it was lower in summer (1.01%) and fall (1.01%) (Table 3). Across all seasons, carcass samples consistently showed higher proportions of positives compared with samples from other sources. By age, ASF prevalence was higher in adults (2.50%) than in piglets (2.07%), while the age‐unknown group showed the highest prevalence overall (4.43%) (Table 3). In summer and fall, prevalence in piglets slightly exceeded that in adults (1.09% vs. 0.71% in summer; 0.91% vs. 0.82% in fall). The age‐unknown group consistently showed the highest prevalence, peaking in spring (6.95%).

Out of 122,078 biological samples, serum samples were available for 17,275 individuals (13.8%), and only two of them tested positive for antibodies to ASF (prevalence 0.01%), one in 2020 and the other in 2021. Both ASF‐seropositive individuals were also positive for ASF virus antigen, and were identified as piglets from the Gangwon (GG) region but from different townships. Each was detected ~40 and 10 days, respectively, after the first ASF‐positive case in the same township.

### 3.3. Logistic Regression Models of Wild Boars Analyzed for ASFV

Multiple logistic regression analyses were conducted to identify factors associated with ASF virus detection in wild boar (Table 4). Among the analyses, two models are presented here: one evaluating the influence of sample source (carcass, hunted, trapped) using all tested samples, and another examining biological variables (sex and age) among samples with complete metadata. In the first model, ASF detection was strongly associated with sample source. Carcass samples were by far the most likely to test positive, with odds ~179 times higher than hunted animals (OR = 178.86, 95% CI: 156.51–204.40, *p* < 0.001). Trapped samples also showed significantly higher odds of ASF detection, being about three times more likely to test positive than hunted animals (OR = 3.02, 95% CI: 2.24–4.06, *p* < 0.001) (Table 4a).

**Table 4:** Logistic regression analyses of factors associated with ASF virus detection in wild boar.

(a) Logistic regression of ASF virus detection by sample source using the full dataset (reference group = hunted)				
Independent variable (sample source)	Coefficient	Std. error	*p*‐Value	Odds ratio (95% CI)
Carcass vs. hunted	5.19	0.07	<0.001	178.86 (156.51–204.40)
Trapped vs. hunted	1.10	0.15	<0.001	3.02 (2.24–4.06)

**(b) Logistic regression of ASF virus detection by sex and age using the complete metadata (reference groups: sex = male, age = piglet)**				
**Independent variable**	**Coefficient**	**Std. error**	***p*-Value**	**Odds ratio (95% CI)**

Sex				
Female vs. male	0.17	0.08	0.04	1.18 (1.01–1.38)
Age				
Adult (>30 kg) vs. piglet	0.24	0.07	0.00	1.27 (1.10–1.46)
Interaction				
Sex × age (female × adult)	0.25	0.10	0.01	1.29 (1.06–1.56)

In the second model, which included only samples with complete metadata on sex and age, both factors were retained as significant predictors (AIC = 20,889; McFadden’s pseudo‐*R* ^2^ = 0.006). The interaction between sex and age significantly improved model fit (*p* = 0.009), indicating that the effect of sex on ASF detection varied by age group. Adult females were more likely to test positive than adult males (OR = 1.29, 95% CI: 1.06–1.56, *p* = 0.009), while overall, females had slightly higher odds of ASF detection than males (OR = 1.18, 95% CI: 1.01–1.38, *p* = 0.041). Adults also had higher odds of infection compared to piglets (OR = 1.27, 95% CI: 1.10–1.46, *p* = 0.001) (Table 4b).

Finally, a logistic regression analysis including only carcass samples, the final selected model retained sine and cosine terms of month and year as predictors (Table 5). Both sine and cosine terms were highly significant (*p* < 0.001), indicating a cyclical pattern in ASF detection, with higher prevalence during winter months and lower prevalence in summer (July, August) (Table 5). The ACF of the model residuals revealed strong autocorrelation at lag 1 (≈ 1 month, ρ ≈ 1.0), which declined sharply by lag 2 (*ρ* ≈ 0.45) and lag 3 (*ρ* ≈ 0.25) (Figure S2). Weak but statistically detectable correlations persisted through lag 4–6, after which no meaningful autocorrelation was observed. The year was also significant (*p* < 0.001), reflecting the increasing overall prevalence from 2020 to 2023.

**Table 5:** Results of logistic regression models for carcass samples only (passive surveillance) (reference year: 2019).

Independent variable		Coefficient	Std. error	*p*‐Value	Odds ratio (95% CI)
Year	2020	1.498	0.157	<0.001	4.47 (3.28, 6.08)
	2021	2.039	0.156	<0.001	7.68 (5.65, 10.44)
	2022	2.097	0.158	<0.001	8.15 (5.97, 11.12)
	2023	1.898	0.169	<0.001	6.67 (4.78, 9.32)
Month^a^	Sine(month)	0.616	0.039	<0.001	—
	Cosine(month)	0.510	0.037	<0.001	—

^a^Month was included using sine and cosine transformations to account for cyclic seasonal variation [18, 19].

### 3.4. Geographical Pattern of ASF Outbreak

**Figure 3:**
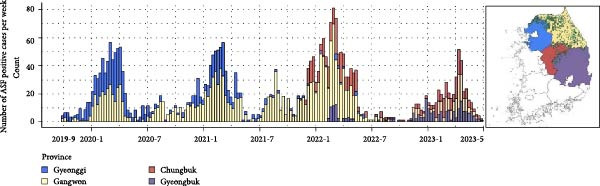
Temporal pattern of ASF epidemic and shift of outbreak region.

**Figure 4:**
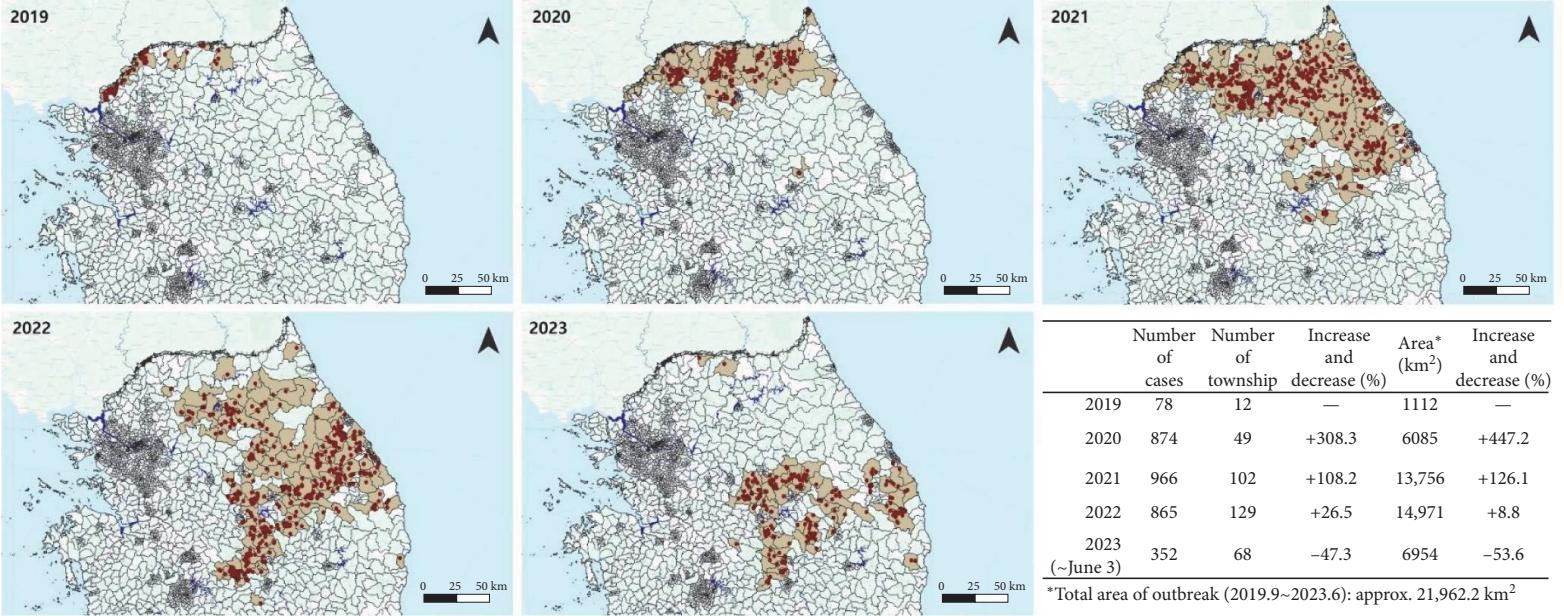
Geographic movement and expansion of regions with wild boar ASF cases.

The first ASF‐positive wild boar was detected within the DMZ, the northernmost part of the ROK (Figures 3 and 4). For nearly 2 years, most cases occurred in the northern Gyeonggi (GG) and GW provinces (Figure 3), initially concentrated in the western and northern parts, until it spread eastward in 2020 and southeastward in 2021, mostly within GW. From 2022 onward, detections in GG regions declined sharply, while new cases started to emerge in Chungbuk (CB) and Gyeongbuk (GB) provinces. GW predominated overall outbreak region until spring 2022, until the abrupt drop of overall positives through the summer (Figure 3). By late 2022, the area around the border of GB and CB province became the main hotspot of ASF cases, although re‐emergence was also observed in GW during the winter of 2022–2023. Overall, the epidemic focus shifted from the northern border to the eastern‐central ROK over 4 years.

**Figure 5:**
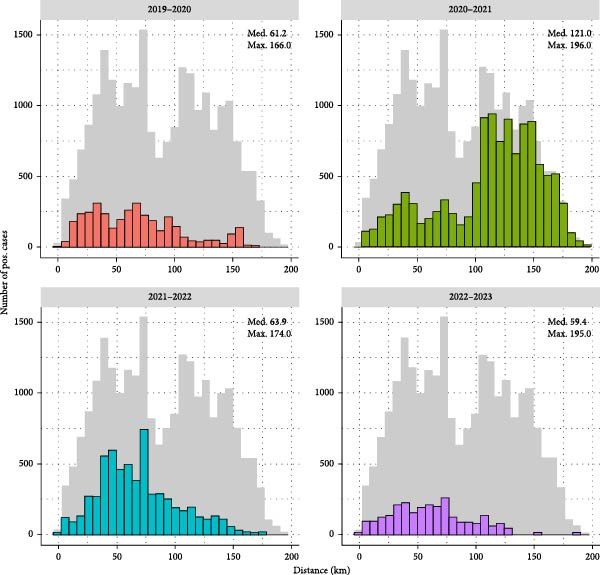
Distribution of distance between ASF positive cases from the last month of two successive years from years 2019–2023.  ^∗^December for year 2019–2022, April and May for the year 2023.

Since the first outbreak, the ASF‐affected area expanded to ~21,962.2 km^2^ within 45 months, based on township units (“eup or myeon”) (Figure 4). The number of affected townships increased from 12 in 2019 to 129 in 2022, while the cumulative area grew from 1112 to 14,971 km^2^ during the same period. The largest increase was observed between 2020 and 2021, with affected townships rising from 49 to 102 (+108%) and area from 6085 to 13,756 km^2^ (+126%). In 2023, the outbreak region contracted to 68 townships covering 6954 km^2^ (–53.6% compared with 2022), mostly confined to the southern part of the previously affected areas. The largest median distance between ASF‐positive cases in the last month of consecutive years was observed between 2020 and 2021, measuring 121 km and corresponding to 10.1 km/month (Figure 5). For other year pairs, estimated monthly movement ranged from 4.9 to 5.3 km/month. These distance‐based estimates were consistent with the area‐based analysis, which showed the largest increase in affected townships and cumulative areas between 2020 and 2021.

**Figure 6:**
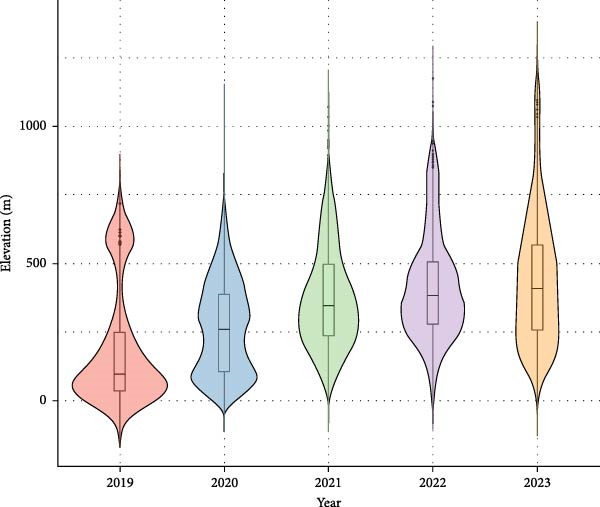
Yearly distribution of elevation of ASF positive cases.

The mean elevation of ASF‐positive wild boar locations (1 km buffer) showed a clear temporal increase during the first 3 years of the epidemic (Figure 6). The mean elevation rose from about 205 m in 2019 to 450 m in 2022, reflecting a progressive shift of outbreaks into more mountainous areas. After 2022, detections occurred at somewhat lower elevations (388 m on average in 2023), but still higher than in the initial phase.

## 4. Discussion

Here, we used comprehensive data on ASF wild boar surveillance to describe the evolution of the disease over time and to gain insight into the overall epidemiological ASF situation in wild boar in the ROK. Unlike other Asian countries where domestic pig farms took a heavy hit from the ASF outbreak, majority of cases in ROK are reported in wild boars. Hence, national control efforts have been focused on thorough wild boar surveillance and population control. During the study period, 365,782 wild boars were eliminated nationwide, and 122,078 wild boar samples were tested for ASF virus. This reflects part of the strenuous effort of the ROK government to control ASF in the wild boar. The tested samples were collected through either active (hunting, trapping) or passive surveillance (carcasses). As in many other countries, the majority of tested samples (92.1%) for ASF were sourced through hunting. While the yearly pattern of total sample size showed a steep increase in the year 2022 due to the implementation of “nationwide surveillance strategy,” the number of carcass samples was unaffected by it and showed the highest number in the year 2020, gradually reducing in successive years till the year 2023. Reduction of carcass samples over the years has been reported in other countries, such as Latvia and Estonia, that experienced a lowering of samples from dead wild boars 3–4 years after the first outbreak [16]. Such a pattern is speculated to be related to the decreased number of wild boars due to geographical expansion of the ASF outbreak throughout the country, along with the implementation of heavy hunting [17]. Nevertheless, this may not fully apply to the situation in the ROK, as ASF is still expanding to new regions, and a reduction in number of hunted wild boars as in the carcass sample has not yet been observed. Instead, the decreasing number of carcasses in the ROK may be associated with other factors. For example, the initial outbreak region of ROK, which was located within and near the DMZ, was mainly composed of rolling hills, compared to the current outbreak region, which is part of the peninsula where the Taebaek Mountain range runs from north to south, with high elevations and rough terrain. Limited access of search personnel in these forests likely impeded the success of finding wild boar carcasses. In addition, despite the consistent expansion of the outbreak region, the number of manpower deployed for passive surveillance was largely cut since 2021 (data not shown), expectedly contributing to the overall decrease of carcass samples. Although hunters are encouraged to search and report any finding of carcasses, their motivation for participating in passive surveillance is well known to be low [20, 21]. Moreover, in ROK, the incentive for finding wild boar carcasses is lower than hunting wild boar, which may have also contributed to losing hunters’ interest in the task.

Seasonally, the number of carcass samples was highest between late winter and spring (February–April), with a rapid drop in summer (less than 20% of spring). A similar pattern has been observed in other countries, where the number of carcass samples was highest during winter through early spring [16]. Accumulation of carcasses within the snow during winter and the increase of agricultural activities, and the thawing of snow in spring likely facilitated the finding of carcasses in this season. Simultaneously, the majority of the outbreak regions in the ROK are mountainous areas, and the thickness of summer vegetation not only limits carcass visibility but also poses safety issues for searching personnel, limiting the efficiency of passive surveillance, which may already be compromised by accelerated speed of carcass decomposition in the season [22].

Wild boar hunting bag size calculated as density has been constant without noticeable change since 2019. Unlike the situation in ROK, reduction of hunting bag size over the years after initial ASF outbreak have been observed in different countries [23, 24], likely related to the geographical expansion of ASF outbreak, as the abundance of wild boar are known to undergo drastic population size reduction (80%–90%) after introduction of ASF, with or without hunting [25]. Since the size of the ASF outbreak region in the ROK accounts for ~22.6% of the total land area, wild boars in yet‐unaffected area likely contributed to the constant hunting bag size during the past 3–4 years. The sharp decrease in the hunting bag size observed for 2023 is presumed to be attributed to the fact that the analysis only included samples collected until 3rd of June, 2023, and spring is the season with the lowest hunting yield.

As expected, ASF prevalence was substantially higher in carcass samples. Due to its virulence, higher chances of finding ASF‐infected individuals among carcasses compared to hunted/trapped wild boars have been universally reported in other countries [24, 26, 27]. Detecting ASF‐positive wild boars from hunting samples is notoriously difficult. In the ROK, this limitation was compounded by the fact that, during the first 2 years of surveillance, only 10% of hunting samples from nonaffected regions were tested. Considering that even in affected areas the prevalence among hunting samples rarely exceeded 1%, this limited surveillance strategy was unlikely to provide meaningful value for early detection. Overall, ASF was far more detectable in carcass samples than in hunting samples. This further highlights the superior utility of carcass‐based surveillance for the timely identification of ASF spread. At the same time, the observed annual increase in ASF prevalence among carcass samples reflects the overall burden of ASF within the wild boar population of the ROK, whereas positive detections from hunting and trapping remained minimal. The continued southward progression of ASF across the Korean Peninsula suggests that reductions in case numbers at earlier outbreak sites are being offset by newly emerging cases in previously unaffected, susceptible regions (Figure 4).

Seasonality was observed in the prevalence of ASF infection, especially for carcasses, which was consistently higher in winter and early spring (December–March), similar to other countries, including Lithuania, Poland, and Germany [4, 22]. This may be due to longer survival of ASF virus in infected carcasses during the winter [22]. Half‐lives of ASF virus inside the host tissue was reported to be over a year at −20°C and a month at +4 °C [28]. Winter temperature in the ROK ranges between 0 and 15°C, which should not only lengthen the survival of the ASF virus but also the duration that a carcass can function as an infection source for susceptible individuals by slowing its decomposition [22]. Temporal autocorrelation analysis further indicates clustering of ASF cases at monthly scales, which may reflect localized infection cycles and delays in carcass detection.

Regarding age, adult individuals constantly showed a higher prevalence than piglets except in summer, presumably due to the high proportion of sounders during this season after the

farrowing [29, 30]. Piglets are not only abundant in their number but tend to stay in groups with a high frequency of direct and indirect contact with their group members, increasing their exposure to ASF infection [23, 30].

When we quantified the spread of ASF through front displacement, ASF in the ROK expanded at about 5–6 km/month in most years. This rate is higher than the slow expansion reported in several European settings, such as Baltic and Polish wild boar populations, where spread was typically <2 km/month (≈0.2–1.5 km/month; [6, 31]). By contrast, our estimates are comparable to those from Lithuania, where expansion reached ≈5 km/month during the first 1–2 years after introduction, before slowing. The somewhat faster progression in Korea may partly reflect the geographical characteristics of the peninsula, which is covered by a high proportion of forests (67.2%), particularly in the eastern regions [8]. Such landscapes may have delayed control responses and allowed ASF to persist longer in the environment [32–35]. While the same rugged and sparsely populated terrain may also have slowed the spread of ASF to some degree, it did not prevent steady expansion through interconnected mountain ranges.

In addition to these geographical influences, another factor likely contributing to the faster spread was the large‐scale regional hunting campaign. This contrasts with many European contexts, where hunting pressure was also intensified but typically applied through more localized and sustained measures aimed at containment. The clear vertical expansion observed in 2021 [36], with short‐term acceleration reaching 22 km/month, likely illustrates the unintended consequences of this large‐scale regional campaign, which operated from December 2020 to March 2021 across five cities/counties previously considered ASF‐free. Heavy hunting, including driven hunt, was promoted in the region with incentives 2.5 times higher than in other regions. However, the distance between outbreak sites and the hunting grounds may not have been sufficient to fully prevent spillover, particularly given the potential influence of driven hunts using dogs on shifting wild boar movement ranges [37, 38]. In addition, considering the large size of these counties (700–1818 km^2^) and the heavily forested landscape, the number of wild boars hunted during 4 months was likely insufficient to suppress ASF spread effectively. Instead, the population disturbance may have facilitated further expansion once the virus invaded [7, 39, 40]. Premature allowance of hunting or delayed ban of hunting in ASF risk regions has been previously pointed out as a potential risk factor for rapid expansion of ASF [13, 41].

To capture spatial dynamics from our data, we applied two complementary approaches: administrative‐unit expansion and direct point‐to‐point distances. Both consistently showed that the most rapid spread occurred between 2020 and 2021, after which the expansion slowed. The distance‐based approach provides a transparent, reproducible measure of yearly displacement, conceptually similar to regression‐based front models that were applied in other front‐wave velocity estimation studies [31, 42].

Finally, altitudinal patterns revealed that ASF detection progressively shifted into higher mountain habitats between 2019 and 2022. Such an upward shift does not necessarily suggest that higher elevations directly favored ASF virus persistence. Instead, it more plausibly reflects the progressive spread of infected wild boar into mountain habitats, where rugged terrain complicates carcass searches. In Korea, this shift appeared as a gradual movement from lowland border areas into upland forested habitats. Reduced detection efficiency at higher elevations may have indirectly influenced the apparent rate of ASF expansion by delaying carcass removal and prolonging local circulation. In this sense, altitude in this context likely influenced surveillance and control capacity while its direct role in ASF ecology remains uncertain. These findings underscore the importance of considering habitat accessibility when interpreting wildlife disease spread.

Lastly, only two seropositive cases were detected during this study period, unlike in other European countries where ASF seropositive animals started to increase within 1–2 years after first outbreak [4, 16]. Constant detection of wild boar carcasses positive for ASFV, with an extremely small number of seropositive, indicates a high case fatality rate of the ASF virus strain currently circulating in the ROK. Accordingly, previous experimental studies reported high pathogenicity and its maintenance in ASF virus isolates from a pig farm in the ROK. Challenged pigs died 8–10 days postinoculation in two consecutive studies, one using ASF strains from 2019 to 2021 and the other from 2022 to 2023 [43, 44]. Therefore, it is presumed that the ASF virus strain that was initially introduced and currently circulating in the ROK has retained its original virulence, and that instances of wild boars surviving infection and subsequently developing a robust antibody response remain exceedingly rare. Nevertheless, among the total number of hunted wild boars, the proportion of samples that also submitted serum is relatively low. Additional efforts, such as thorough education, would be required to increase the participation of hunters to collect and submit fresh serum samples for antibody testing.

Despite extensive nationwide control measures, ASF has continued to spread southward across the Korean Peninsula. Refinement and reinforcement of control strategies, guided by the findings of this study, may improve the response to the evolving epidemiological situation. In particular, securing sufficient carcass search personnel in affected regions, while considering local demographic and landscape factors, should be prioritized. Resources for ASF control would be most effectively allocated to strengthening passive surveillance at outbreak sites [45], rather than incentivizing hunting of apparently healthy wild boars in areas far from ASF activity. The nationwide year‐round bounty system for wild boar population reduction has been implemented for the past 4 years. However, the long‐term efficiency of intensive hunting over broad areas remains questionable, especially for prolific species such as wild boar [45–49], and may inadvertently encourage illegal practices [50], increasing biosecurity risks that may contribute to long‐distance “jumps” of ASF [12]. The contribution of hunting to ASF control may be more effective when efforts are concentrated within buffer zones adjacent to outbreak areas, where targeted activities are likely to better restrict further spread of the virus, rather than being diffusely applied nationwide [51, 52]. Lastly, hunters exert a considerable influence on ASF outbreaks and their control. Future studies investigating hunters’ perceptions of ASF epidemiology and their views on applied control and biosecurity measures would provide critical insights to promote good practices and counteract demotivation, ultimately improving the overall effectiveness of ASF management.

This study is essentially descriptive in nature, summarizing surveillance outputs and observed epidemiological patterns of ASF in wild boars in the Republic of Korea. While this limits the potential for causal inference or mechanistic modeling, such descriptive reporting provides a unique national‐scale overview. These results offer a foundation for future in‐depth spatial and risk factor analyses that are needed to better understand ASF dynamics.

## Conflicts of Interest

The authors declare no conflicts of interest.

## Funding

This research was funded by the National Institute of Environmental Research (NIER) (Grant 2019‐01‐01‐006) and the National Institute of Wildlife Disease Control and Prevention (NIWDC) (Grant 2021‐001, 2022‐001), both under the Ministry of Environment, Republic of Korea.

## Supporting Information

Additional supporting information can be found online in the Supporting Information section.

## Supporting information


**Supporting Information** Table S1: Annual and monthly counts of surveillance samples. Table S2: Distribution of surveillance samples by source, with proportions independently stratified by age and sex. Figure S1: Distribution of township (‘eup’ or ‘myeon’) areas in the ROK. Figure S2: Autocorrelation function (ACF) and partial autocorrelation function (PACF) of Pearson residuals from the carcass‐based GLM of ASF virus detection. Figure S3: Weekly pattern of positive detection cases from different sources. Figure S4: Histogram of pair‐wise distance between ASF positive points at the last months of each year.

## Data Availability

The data that support the findings of this study are available from the corresponding author upon reasonable request.
