# Supplementary material for: Exploratory Report of Wild Boar Surveillance and Epidemiological Course of African Swine Fever Outbreak; Case in the Republic of Korea From 2019 to 2023
Source: Transbound Emerg Dis. 2025 Dec 4;2025:3538366. doi: 10.1155/tbed/3538366 (PMC12707299; doi:10.1155/tbed/3538366)

**Supporting information**

Table S1 Annual and monthly counts of surveillance samples

| Month  Year | Jan. | Feb. | Mar. | Apr. | May | Jun. | Jul. | Aug. | Sep. | Oct. | Nov. | Dec. | **Total** |
| --- | --- | --- | --- | --- | --- | --- | --- | --- | --- | --- | --- | --- | --- |
| 2019 | - | - | - | - | - | - | - | - | 96 | 1,067 | 1,912 | 1,685 | **4,760** |
| 2020 | 1,231 | 1,291 | **1,443** | 1,157 | 850 | 856 | **1,391** | 602 | 503 | 675 | 942 | **1,420** | **12,361** |
| 2021 | 1,334 | 1,636 | 1,467 | 919 | 716 | 854 | **2,262** | 1,656 | 1,042 | **1,734** | **1,978** | 1,538 | **17,136** |
| 2022 | 1,584 | 2,141 | 2,986 | 2,542 | 2,599 | 4,309 | **9,681** | 8,035 | 6,440 | **8,289** | **10,526** | 6,653 | **65,785** |
| 2023 | 4,886 | 5,150 | 4,985 | 3,484 | 3,196 | 335 | - | - | - | - | - | - | **22,036** |
| **Total** | **9,035** | **10,218** | **10,881** | **8,102** | **7,361** | **6,354** | **13,334** | **10,293** | **8,081** | **11,765** | **15,358** | **11,296** | **122,078** |

Table S2 Distribution of surveillance samples by source, with proportions independently stratified by age and sex

| Factors  Source | Female | Male | Unknown | Piglet | Adult | Unknown | **Total** |
| --- | --- | --- | --- | --- | --- | --- | --- |
| Carcass | 2,491  (25.7%) | 1,966  (20.3%) | 5,228  (54.0%) | 2,162  (22.3%) | 3,293  (34.0%) | 4,230  (43.7%) | **9,685** |
| Hunted | 50,163  (47.9%) | 50,415  (48.2%) | 4,041  (3.9%) | 36,186  (34.6%) | 60,214  (57.6%) | 8,219  (7.9%) | **104,619** |
| Trapped | 3,637  (46.8%) | 3,370  (43.3%) | 767  (9.9%) | 2,628  (33.8%) | 4,279  (55.0%) | 867  (11.2%) | **7,774** |
| **Total** | **56,291**  **(46.1%)** | **55,751**  **(45.7%)** | **10,036**  **(8.2%)** | **40,976**  **(33.6%)** | **67,786**  **(55.5%)** | **13,316**  **(10.9%)** | **122,078** |

Figure S1. Distribution of township (‘eup’ or ‘myeon’) areas in the ROK

(A) Histogram on a linear scale, showing a right-skewed distribution with most values clustered at the lower end. (B) Histogram on a logarithmic scale, highlighting that more than half of townships were between 1 and 30 km² and only a few mountainous units exceeded 70 km² (n = 5,047).

(A) Histogram on a linear scale


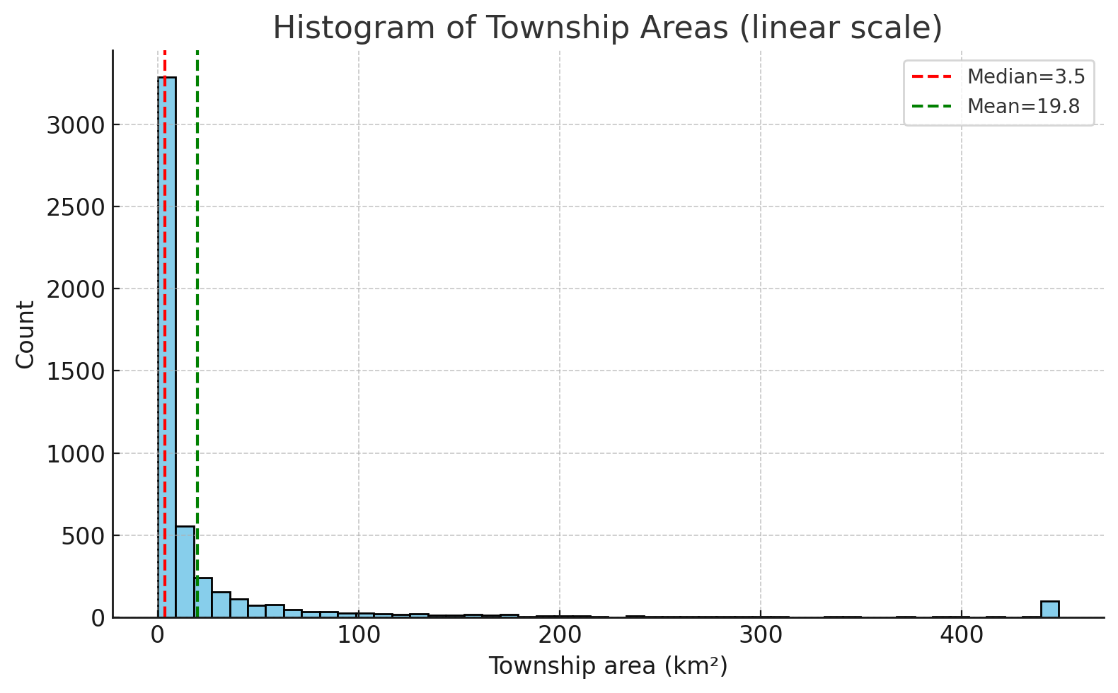


(B) Histogram on a logarithmic scale


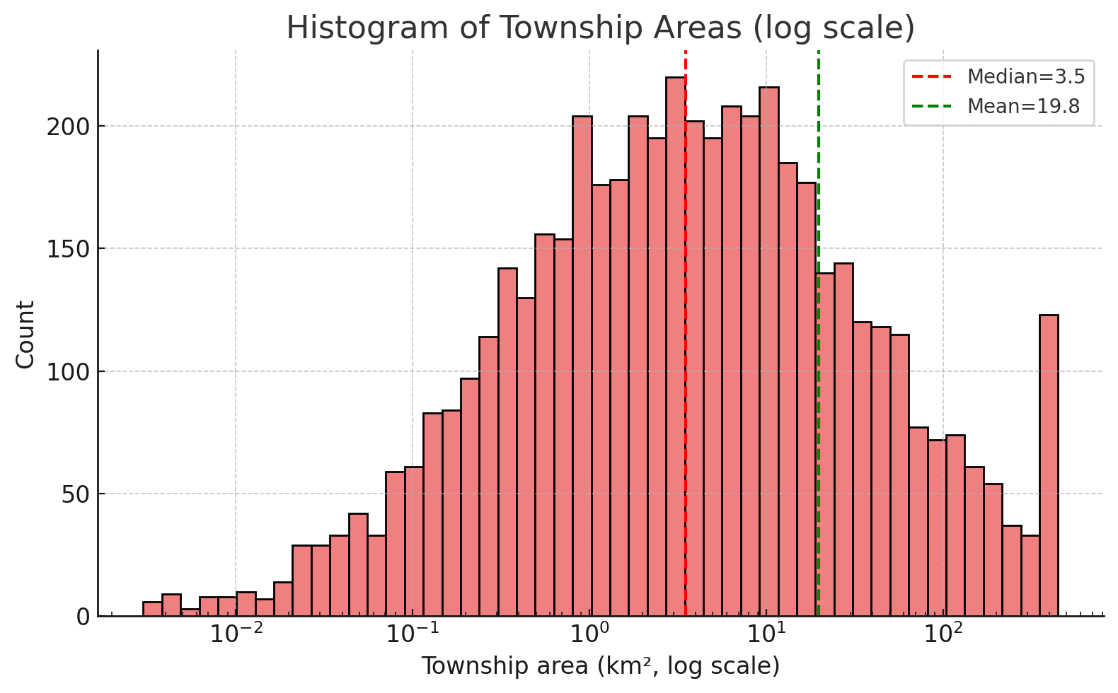


Figure S2. Autocorrelation function (ACF) and partial autocorrelation function (PACF) of Pearson residuals from the carcass-based GLM of ASF virus detection


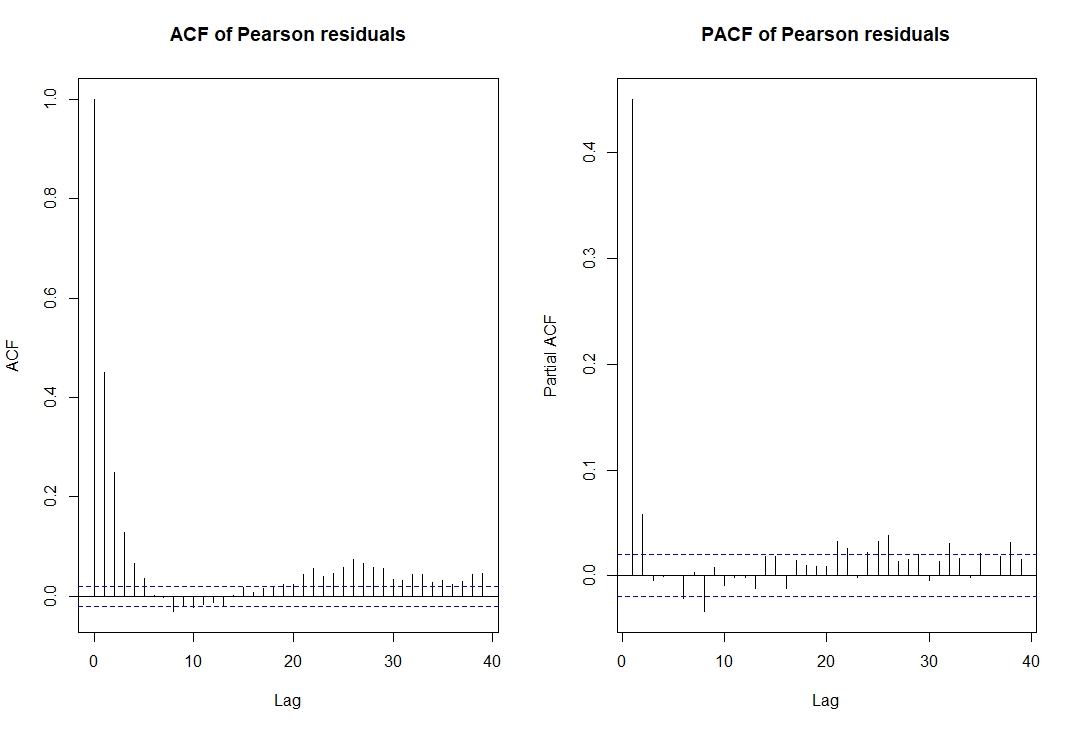


Figure S3. Weekly pattern of positive detection cases from different sources


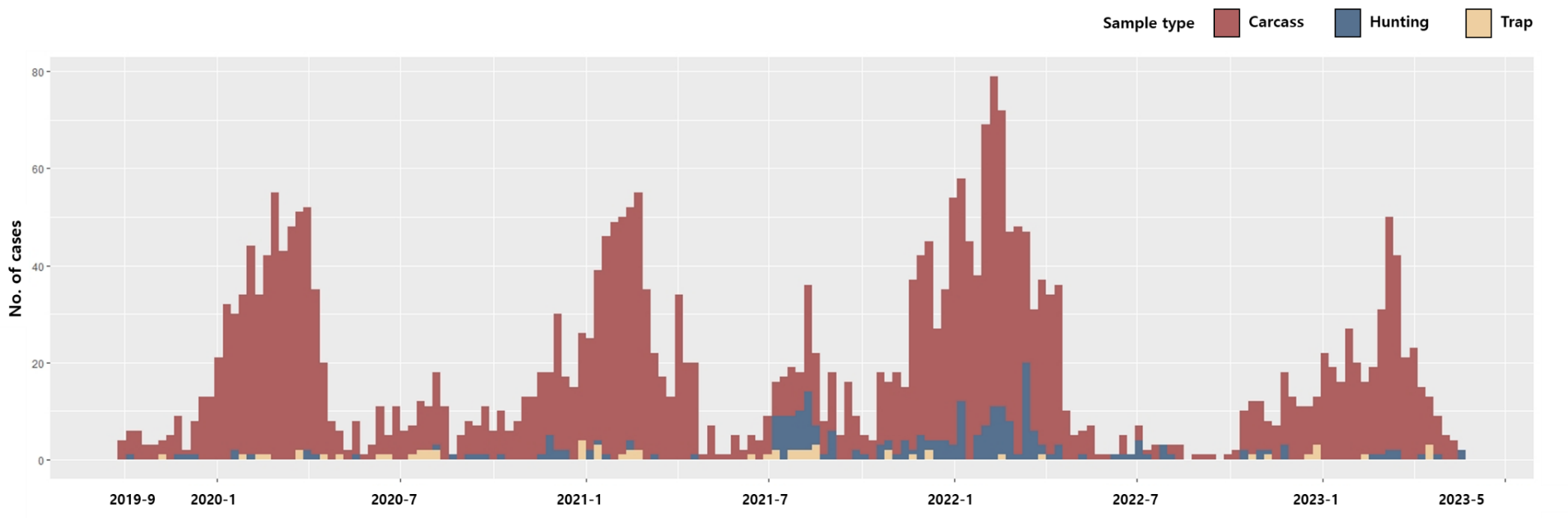


Figure S4. Histogram of pair-wise distance between ASF positive points at the last months of each year


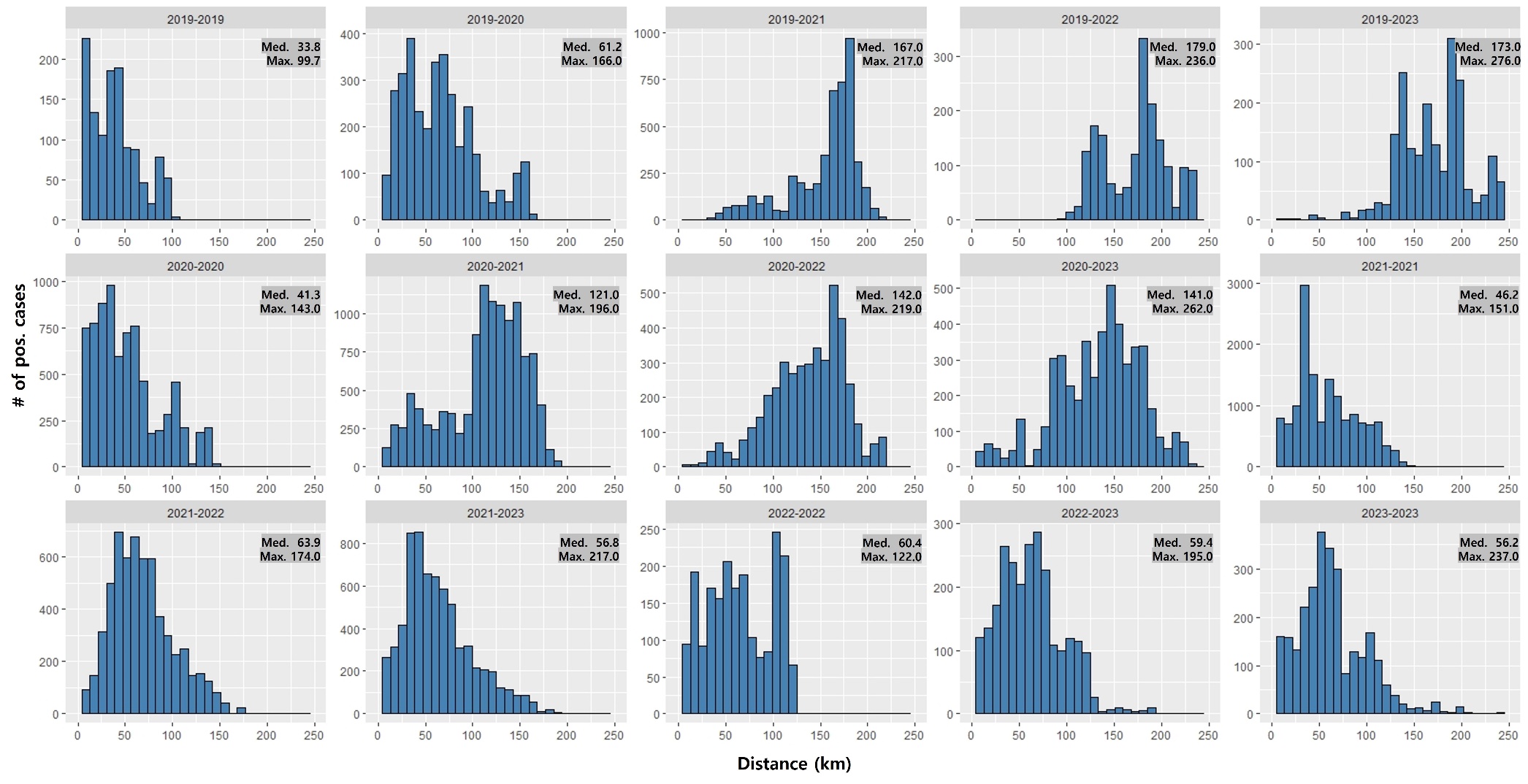

Supplement: Supplementary file 1 — Supporting Information Table S1: Annual and monthly counts of surveillance samples. Table S2: Distribution of surveillance samples by source, with proportions independently stratified by age and sex. Figure S1: Distribution of township (‘eup’ or ‘myeon’) areas in the ROK. Figure S2: Autocorrelation function (ACF) and partial autocorrelation function (PACF) of Pearson residuals from the carcass‐based GLM of ASF virus detection. Figure S3: Weekly pattern of positive detection cases from different sources. Figure S4: Histogram of pair‐wise distance between ASF positive points at the last months of each year. [file TBED-2025-3538366-s001.docx]
